# Intranasal vs. intramuscular administration of azaperone, midazolam and ketamine in pigs

**DOI:** 10.3389/fvets.2024.1408103

**Published:** 2024-09-25

**Authors:** Isabela Peixoto Rabelo, Cinthya de Andrade Gujanwski, Inácio Silva Viana, Vanessa Barroco de Paula, Ariadne Rein, Sara Peixoto Rabelo, Carlos Augusto Araújo Valadäo

**Affiliations:** ^1^Department of Veterinary Internal Medicine and Surgery, School of Agricultural and Veterinarian Sciences, São Paulo State University (Unesp), Jaboticabal, SP, Brazil; ^2^Faculdade Ciências Médicas de Minas Gerais, Belo Horizonte, MG, Brazil

**Keywords:** tranquilizers, benzodiazepines, chemical restraint, animal model, anesthesia

## Abstract

**Objective:**

To compare the efficacy of intranasal (IN) and intramuscular (IM) administrations of azaperone (3 mg kg^−1^), midazolam (0. 3 mg kg^−1^), and ketamine (7 mg kg^−1^) combination (AMK) in pigs. Study design: Randomized clinical trial. Animals: sixteen adult male pigs, immunocastrated, of mixed lineage.

**Methods:**

In phase I, these animals were randomly assigned to intranasal (GIN, *n* = 8) and intramuscular (GIM, *n* = 8) groups for arterial blood sample collection at 10, 20, 30, 45, 60, and 90 min after AMK administrations for gas and electrolyte analysis. In phase II, performed 1 week after phase I, the 16 pigs were allocated to both groups (GIM, *n* = 16/GIN, *n* = 16) and submitted to the same chemical restraint (CR) protocol, with a 96-h interval between administrations. Behavioral parameters (degree of CR, muscle relaxation, loss of postural reflex, and sound stimulus response) and vital parameters (pulse rate, respiratory rate, oxygen saturation, and rectal temperature) were evaluated after recumbency (Trec) and at 5, 15, 30, 45, 60, and 90 min after administrations. In addition, the latency period and duration of CR were determined.

**Results:**

Latency to recumbency and duration of CR in GIN were shorter. CR scores did not vary between groups. Muscle relaxation was more intense in GIN at Trec. An initial tachycardia was observed, followed by a reduction in heart rate from T5 to T90 in both treatments (*p* < 0.05). The respiratory rate was higher at T45, T60, and T90 in GIN compared to baseline. Rectal temperature reduced in GIM from T45 onwards. ${\text{PaCO}}_{2}^{\text{t}}$ elevated at T90 in the GIM (*p* < 0.05) and there was an incidence of mild hypoxemia in 47% of the animals in the GIM.

**Conclusions and clinical relevance:**

IN administration was as effective as IM administration in promoting safe chemical restraint, with minimal changes in homeostasis, with a shorter duration and latency period.

## 1 Introduction

The advancement of therapeutic medicine has been greatly facilitated by the utilization of animal models to investigate the etiopathogenesis and pathophysiological mechanisms underlying various diseases. Due to their abundant anatomical, physiological, and genomic similarities with humans, pigs have become a preferred model for investigating cardiovascular (1–3), skeletal (4, 5), urinary (6) systems, as well as metabolic, neurodegenerative, and genetic diseases (7). In comparison to primates, pigs exhibit short generation intervals, rapid growth rates, prolific litters, and standardized breeding techniques (8).

Ensuring the wellbeing of animals involved in biomedical studies is the responsibility of the researcher and is essential for obtaining reliable results (9). Pigs are poorly tolerant of physical restraint, which can induce metabolic changes such as hyperthermia and respiratory distress (10). In this context, chemical restraint, while reducing stress, can be associated with local anesthesia for surgical purposes and offers certain advantages over general anesthesia, including easier administration, lower cost, reduced equipment requirements, shorter recovery time, and lower risk of complications (11).

The combination of azaperone, midazolam, and ketamine was chosen for this study due to their complementary effects and suitability for use in swine. Azaperone provides reliable tranquilization and muscle relaxation, making it effective for procedures requiring immobilization (12–14). Midazolam enhances sedation, promotes muscle relaxation, reduces the requirement for other anesthetics, and mitigates the unwanted effects of dissociative agents (15, 16). Ketamine is considered a safe anesthetic agent due to its stimulating effects on the cardiovascular system (17, 18) and minimal respiratory depression (19). Additionally, ketamine offers dissociative anesthesia with a rapid onset of action and a short duration, which is beneficial for quickly achieving and maintaining the desired anesthetic state (16). These properties contribute to its broad dosage range. In swine, the recommended doses for these three drugs 1–4 mg/kg of azaperone (20, 21), 0.2–0.4 mg/kg of midazolam (22), and 1–20 mg/kg of ketamine (21, 23).

The intramuscular (IM) route has been widely used in swine for the administration of sedatives and tranquilizers. However, there is the potential risk of muscle damage and extended recovery time due to drug deposition in muscle tissue (10, 20, 24). On the other hand, intravenous (IV) administration is challenging in conscious animals, as venous access requires forceful physical restraint (11).

Intranasal (IN) administration is a painless and less invasive method (25), with drug absorption facilitated by the olfactory and trigeminal neural pathways, bypassing the blood-brain barrier (BBB) (26). The use of this route for administering sedatives, tranquilizers and analgesics has been described in several species, including humans and pigs (10, 13, 27–29).

Therefore, this study aimed to propose a less invasive alternative for chemical restraint (CR) in pigs, with a focus on maintaining homeostasis by minimizing the stress associated with physical restraint.

## 2 Materials and methods

### 2.1 Animals and housing

The trial was carried out at the Swine Studies Laboratory (LabSui) of the Animal Science Department at São Paulo State University (Unesp), School of Agricultural and Veterinarian Sciences (FCAV), Jaboticabal-SP, Brazil. This study was approved by the Ethics Committee on the Use of Animals (CEUA) of FCAV/Unesp/Jaboticabal (protocol 2296/2021). Sample size determination followed the formula proposed by the “Guidelines for the care and use of mammals in neuroscience and behavioral research” (30), with sixteen adult pigs utilized (assuming two groups, α = 0.05, and 1 – β = 0.8). The subjects were immunocastrated males, of mixed lineage (Landrace x Large White), with an average weight of 102 ± 12 kg.

The pigs were housed in individual pens (2.7 m^2^) with a fully slatted plastic floor and controlled room temperature (21°C−27°C). They had access to water through nipple-type drinkers and were provided with feed (semi-automatic trough feeder) *ad libitum*. The diet consisted primarily of corn and soy, with nutritional content comprising 10.96% crude protein, 0.66% digestible lysine, 0.500% calcium, 0.230% phosphorus, and 2,525 Kcal Kg^−1^ net energy. The health of the animals was confirmed through both clinical and laboratory examinations. Prior to the CR protocol, the pigs underwent a solid food fast (6–8 h) while maintaining free access to water. Following the completion of the longest washout period (equivalent to 10 half-lives of ketamine), the animals were sent to slaughter.

### 2.2 Experimental design and group formation

The experiment was carried out in two stages. In Phase I, conducted over 5 days (D1 to D5), the 16 pigs were allocated into 2 groups (*n* = 8) and received a combination of 3 drugs (AMK): 3 mg kg^−1^ of azaperone (Destress^®^ Desvet, SP, Brazil), 0.3 mg kg^−1^ of midazolam (Dormire^®^ Cristália, SP, Brazil), and 7 mg kg^−1^ of ketamine (Ketalex^®^ Dechra, PR, Brazil). One group received the AMK combination intranasally (GIN, *n* = 8) and the other intramuscularly (GIM, *n* = 8). In this phase, arterial blood samples were collected for the analysis of gases and electrolytes. The distribution of animals into groups was done by ordering them by increasing weight and pairing them two by two, followed by eight draws until the groups were filled. This method was employed to reduce weight discrepancies between the GIN and GIM. In Phase II, conducted between D7 and D13, animals from both groups (GIN and GIM, *n* = 16) underwent the same CR protocol as described in Phase I, with a 96-h interval between administrations. This phase aimed to assess clinical and behavioral parameters. The two-stage assay was necessary to avoid additional stress from manipulation during Phase I, which could affect the behavioral parameters evaluated in Phase II. A 96-h interval was established, approximately twice the “washout” period of the longest-acting drugs (ketamine and midazolam), to ensure complete elimination of these substances before the next administration. Administrations were performed between 8:00 and 12:30 h to avoid the hottest periods of the day. In Phase II, two animals were treated simultaneously every 1.5 h, totaling 6 animals per day. A consistent time was maintained for administration in each pig, for example, animal 1 received AMK at 8:00 h in GIM and at the same time when integrated into GIN.

### 2.3 Intranasal and intramuscular administration

In both phases, restraint for the administration of the AMK combination was achieved using a snubbing rope, positioned behind the pig's tusks for a brief period (30–60 seconds). Administration via the intranasal route (GIN) was carried out over 20 seconds, exclusively in the right nostril, using a 20 mL disposable syringe. The total volume of AMK (21 ± 3.5 mL) was divided into two syringes, when necessary, and its administration was guided by a 14 G × 45 mm polytetrafluoroethylene catheter. The pigs' snouts were elevated for 30 seconds. In GIM, the AMK combination was applied intramuscularly to the middle third of the neck. This was achieved using appropriate size syringes and 40 × 1.2 mm disposable needles after skin antisepsis with a surgical pad soaked in 70% alcohol.

### 2.4 Arterial blood collection (phase I)

The ears were cleansed with 70% alcohol, followed by the application of topical lidocaine 10% (Xylestesin^®^ Cristália, SP, Brazil) for skin desensitization. After administration of the AMK combination, a 22G peripheral catheter (Descarpack^®^ SP, Brazil) was inserted into the auricular artery for sample collection at T10, T20, T30, T45, T60, and T90 (each subscript number corresponds to the moment, in minutes, counted after administration). Syringes containing lytic heparin (A-Line 3 mL SAG; BD™; MG, Brasil) were used and these samples were processed immediately using a blood gas analyzer (cobas b 123 POC system; Roche, SP, Brazil). The measured parameters included pH, ${\text{PaCO}}_{2}^{\text{t}}$, ${\text{PaO}}_{2}^{\text{t}}$, base excess (BE), anion gap (AG), and concentrations of Na^+^, K^+^, Ca^2+^, Cl^−^, ${\text{HCO}}_{3}^{-}$, glucose, and lactate.

### 2.5 Physiological and behavioral parameters (phase II)

Assessments were conducted at recumbency (Trec), T5, T10, T15, T30, T45, T60, and T90 following the administration of AMK. Physiological parameters, including pulse rate (PR) and oxyhemoglobin saturation (SpO_2_), were evaluated using pulse oximetry (CONTEC08C; Contec™, Hebei, China); respiratory rate (*f*_R_) was assessed by observing rib cage movements, and rectal temperature (Trectal) was measured using a digital thermometer. Behavioral parameters were evaluated during the same periods, extending until the animal remained recumbent, as detailed in Table 1. In addition, the latency period and duration of CR were determined. The latency period was defined as the time elapsed until decubitus occurred following the administration of AMK. The duration of CR was documented from the onset of recumbency until the animal returned to the quadrupedal position. Due to the small size of the team conducting the trial, a double-blind evaluation was not feasible.

**Table 1:** Numerical scale used to evaluate behavioral responses in pigs.

**Parameter**	**Score**	**Observation**
Chemical restraint	0	Absent
	1	Mild (lowers head, reduced eyelid reflex, refuses to lie down)
	2	Moderate (supports the head, sternal or lateral decubitus)
	3	Deep (accepts supine position, eyeball rotated)
Muscle relaxation	0	Normal limb muscle tone
	1	Soft (resistance to limb bending)
	2	Moderate (decreased resistance to limb flexion)
	3	Deep (limb flexion without resistance)
Posture	0	Quadrupedal position
	1	Ataxia or sitting
	2	Sternal recumbency
	3	Lateral recumbency
Noise response^*^	0	Normal
	1	Light (moves ears/eyes and body)
	2	Moderate (moves ears and/or eyes, does not move body)
	3	Deep (no response)

^*^Claps repeated 3 times at 30 cm from the ear. Cornick and Hartsfield (44); adapted to the swine species (45) and readapted in the present study.

### 2.6 Statistics

All analyzes were performed using the Graphpad Prism program (version 9.0.0. 121). Normality of variables pertaining to blood gas analysis, vital parameters, latency and duration of CR was confirmed by the Shapiro-Wilk test. For comparison between groups, the *T* test was applied to gasometric variables, while physiological variables, latency and duration of CR were evaluated using the paired *T* test. To investigate variations over time, Repeated Measures Analysis of Variance (RM-ANOVA) was used. Additionally, the Dunnett's test was used to identify any differences between the first collection/evaluation moment and subsequent ones. The behavioral variables expressed in scores were subjected to the Friedman test, followed by Dunn, to analyze variations over time, and the Wilcoxon test, aiming to detect differences between groups. Concerning missing data, they were more prevalent in the later time points, particularly in GIN, as animals in this group exhibited quicker recovery, and data were only collected while the animals were in recumbency. Consequently, comparisons between groups were feasible until T90.

## 3 Results

### 3.1 Considerations

The trial began with a total of 20 animals. One of them showed signs of prostration on the 2^nd^ day (D2) and, after clinical and laboratory evaluation, was removed from the trial to receive the necessary care and did not receive the AMK combination. During phase II, another animal reacted with head movements during intranasal administration and was removed from the evaluations. In general, all administrations were carried out uneventfully, and all animals were recumbent after administration of AMK.

### 3.2 Characteristics of the chemical restraint

The latency period was shorter in GIN (GIN: 63 ± 47 seconds; GIM: 113 ± 39 seconds; *p* = 0.002). GIN also exhibited a shorter duration of CR (119 ± 54 min) compared to GIM (163 ± 47 min; *p* = 0.0015). CR scores remained consistent between groups and times, with an overall median of three. Intranasal administration induced immediate muscle relaxation (Trec: GI*N* = 3 vs. GIM = 1; *p* = 0.008). In GIM, maximum muscle relaxation occurred at T15, T30, and T45, compared to baseline (*p* = 0.0015). Scores for posture and response to sound did not vary between groups and times.

### 3.3 Physiological and gasometric parameters

The assessed vital parameters (PR, *f*_R_, SpO_2_, and Trectal) are presented in Table 2 (mean values ± standard deviation) and Table 3 (percentage representation of alterations). A reduction in PR was observed in both groups relative to baseline (*p* < 0.05). Table 3 indicates that most animals exhibited tachycardia in Trec. GIN displayed a higher percentage of tachycardia at T45, T60, and T90 (Table 3), differing from GIM (T45: *p* = 0.0288; T60: *p* = 0.0127; T90: *p* = 0.0203).

**Table 2:** Values (mean ± standard deviation) of pulse rate (PR), respiratory rate (*f*_R_), oxygen saturation (SpO_2_), and rectal temperature (T_rectal_) of adult pigs, which received a combination of azaperone (3 mg kg^−1^), midazolam (0.3 mg kg^−1^) and ketamine (7 mg kg^−1^), via intramuscular (GIM, *n* = 16) and intranasal (GIN, *n* = 16) routes.

**Variable (unit)**	**Group**	**Timepoints**						
		**T**_rec_	${\text{T}}_{5}^{*}$	**T**_15_	**T**_30_	**T**_45_	**T**_60_	**T**_90_
PR (bpm)	GIM	120 ± 18	94 ± 16^†^	72 ± 16^†^	78 ± 12^†^	83 ± 14^†^	84 ± 16^†^	84 ± 14^†^
	GIN	119 ± 12	93 ± 25^†^	81 ± 21^†^	81 ± 18^†^	94 ± 16^†Δ^	95 ± 26^†Δ^	100 ± 14^†Δ^
*f*_R_ (mpm)	GIM	33 ± 11	34 ± 13	28 ± 8	32 ± 11	35 ± 17	37 ± 16	42 ± 25
	GIN	31 ± 7	30 ± 8	33 ± 11	38 ± 14	43 ± 15^†^	50 ± 18^†^	57 ± 19^†^
SpO_2_ (%)	GIM	96 ± 2	96 ± 2	96 ± 2	96 ± 2	96 ± 2	96 ± 3	97 ± 1
	GIN	94 ± 3^Δ^	95 ± 3	96 ± 3	95 ± 4	97 ± 2	96 ± 3	96 ± 3
T_rectal_ (°C)	GIM	38.4 ± 0.5	38.3 ± 0.6	38.2 ± 0.5	38.0 ± 0.5	37.9 ± 0.5^†^	37.8 ± 0.5^†^	37.8 ± 0.6^†^
	GIN	38.5 ± 0.5	38.5 ± 0.4	38.5 ± 0.5^Δ^	38.4 ± 0.6^Δ^	38.5 ± 0.6^Δ^	38.2 ± 0.6^Δ^	38.6 ± 0.7^Δ^

^†^Different from Trecumbency (T_rec_), in the same group, by Dunnett's test (*p* < 0.05). ^Δ^Different from GIM, by paired *T* test (*p* < 0.05). ^*^Minutes after AMK administration.

**Table 3:** Percentage representation of pulse rate (PR), respiratory rate (*f*_R_), oxygen saturation (SpO_2_), and rectal temperature (T_rectal_) of adult pigs, which received a combination azaperone (3 mg kg^−1^), midazolam (0.3 mg kg^−1^), and ketamine (7 mg kg^−1^), via intramuscular (GIM, *n* = 16) and intranasal (GIN, *n* = 16) routes.

**Variable**	**Timepoints**	**GIN (% of animals)**			**GIM (% of animals)**			**Reference (unit)**
		**Below**	**Normal**	**Above**	**Below**	**Normal**	**Above**	
PR	T_rec_	0	0	100	0	6	94	^a^ 75–89 (bpm)
	${\text{T}}_{5}^{*}$	19	25	56	13	38	50	
	T_15_	38	38	25	63	31	6	
	T_30_	56	13	31	31	63	6	
	T_45_	14	14	71	25	63	13	
	T_60_	0	42	58	25	38	38	
	T−90	0	27	73	6	38	56	
	**Mean**	**18**	**23**	**59**	**23**	**39**	**38**	
SpO_2_	T_rec_	25	75	-	6	94	-	^b^ 92–100 (%)
	${\text{T}}_{5}^{*}$	19	81	-	0	100	-	
	T_15_	13	88	-	6	94	-	
	T_30_	13	88	-	0	100	-	
	T_45_	0	100	-	0	100	-	
	T_60_	17	83	-	0	100	-	
	T_90_	18	82	-	0	100	-	
	**Mean**	**15**	**85**	-	**2**	**98**	-	
*f*_R_	T_rec_	0	63	38	0	63	38	^c^ 16–35 (mpm)
	${\text{T}}_{5}^{*}$	0	69	31	0	56	44	
	T_15_	0	56	44	13	75	13	
	T_30_	0	56	44	0	75	25	
	T_45_	0	29	71	0	81	19	
	T_60_	0	25	75	0	63	38	
	T_90_	0	18	82	0	50	50	
	**Mean**	**0**	**45**	**55**	**2**	**66**	**32**	
T_rectal_	T_rec_	0	100	0	0	100	0	^d^ 37.0–39.6 (°C)
	${\text{T}}_{5}^{*}$	0	100	0	6	94	0	
	T_15_	0	100	0	0	100	0	
	T_30_	0	100	0	6	94	0	
	T_45_	0	100	0	6	94	0	
	T_60_	0	100	0	6	94	0	
	T_90_	0	91	9	13	88	0	
	**Mean**	**0**	**99**	**1**	**5**	**95**	**0**	

^a^PR: 81–89 bpm (33); 75–85 bpm (34). ^b^SpO_2_: 97–100 % (35); 92–95 % (36). ^c^*f*_R_: 16–25 mpm (36); 25–35 mpm (34). ^d^T_rectal_: 37–39.6°C (36); 38.5–39.1°C (34). ^*^Minutes after AMK administration. T_rec_: Trecumbency.

In GIN, *f*_R_ increased at T45 (*p* = 0.0312 and 71% of tachypnea), T60 (*p* = 0.0144 and 75% of tachypnea), and T90 (*p* = 0.0051 and 82% of tachypnea), compared to baseline (Trec). SpO_2_ remained constant across all times, but 25% of GIN animals exhibited reduced SpO_2_ values at Trec, differing from GIM (*p* = 0.0364). In GIM, Trectal reduced relative to baseline at T45, T60, and T90 (*p* < 0.05) and compared to GIN from T15 onwards. In GIN, 99% of animals remained within the normothermia range (Table 3). On the other hand, 5% of the GIM animals showed a drop in body temperature during the CR period.

Gasometric parameters are presented in Table 4 (average values ± standard deviation) and Table 5 (percentage representation of changes). ${\text{PaCO}}_{2}^{\text{t}}$ decreased at T90 in GIM compared to T10 (*p* = 0.0139) but remained constant across groups, averaging 45 ± 6 mmHg throughout. In GIN, an incidence of hypocapnia (${\text{PaCO}}_{2}^{\text{t}}$ < 35 mmHg) was identified in 14% and 20% of animals at T60 and T90 (Table 5), respectively. ${\text{PaO}}_{2}^{\text{t}}$ values did not demonstrate significant variations between groups and moments (*p* > 0.05). However, an average of 11% of the animals in the GIN and 47% of the animals in the GIM had ${\text{PaO}}_{2}^{\text{t}}$ values below 82 mmHg (Table 5).

**Table 4:** Values (mean ± standard deviation) of gasometric parameters of adult pigs, which received a combination of azaperone (3 mg kg^−1^), midazolam (0.3 mg kg^−1^), and ketamine (7 mg kg^−1^), via intramuscular (GIM, *n* = 8) and intranasal (GIN, *n* = 8) routes.

**Variable (unit)**	**Group**	**Timepoints**					
		${\text{T}}_{10}^{*}$	**T**_20_	**T**_30_	**T**_45_	**T**_60_	**T**_90_
pH_st_	GIM	7.48 ± 0.04	7.47 ± 0.03	7.48 ± 0.03	7.48 ± 0.03	7.48 ± 0.04	7.49 ± 0.03
	GIN	7.48 ± 0.03	7.49 ± 0.03	7.48 ± 0.03	7.48 ± 0.04	7.48 ± 0.04	7.47 ± 0.04
${\text{PaCO}}_{2}^{\text{t}}$ (mmHg)	GIM	47 ± 7	46 ± 7	48 ± 5	46 ± 5	44 ± 4	43 ± 5^†^
	GIN	50 ± 8	45 ± 5	43 ± 5	44 ± 6	42 ± 6	40 ± 8
${\text{PaO}}_{2}^{\text{t}}$ (mmHg)	GIM	78 ± 6	80 ± 8	81 ± 7	80 ± 11	86 ± 9	97 ± 17
	GIN	76 ± 9	86 ± 9	102 ± 33	82 ± 2	97 ± 3	102 ± 3
${\text{cHCO}}_{3\text{st}}^{-}$ (mMol L^−1^)	GIM	28.8 ± 2.4	28.2 ± 2.2	28.6 ± 2.1	28.5 ± 2.2	28.5 ± 2.4	29.2 ± 1.9
	GIN	29.0 ± 2.1	29.3 ± 1.9	29.0 ± 2.2	29.0 ± 2.5	28.7 ± 2.6	27.8 ± 2.6
BE (mMol L^−1^)	GIM	5.5 ± 2.9	4.7 ± 2.6	5.1 ± 2.5	5.1 ± 2.6	5.0 ± 2.9	5.8 ± 2.3
	GIN	5.7 ± 2.5	5.9 ± 2.3	5.5 ± 2.6	5.6 ± 3.0	5.2 ± 3.1	4.1 ± 3.1
AG (mMol L^−1^)	GIM	7.5 ± 4.1	7.6 ± 2.5	6.8 ± 2.2	7.4 ± 2.4	7.8 ± 2.2	8.6 ± 2.2
	GIN	8.1 ± 2.7	8.1 ± 2.3	7.6 ± 2.2	8.2 ± 3.3	8.3 ± 3.7	9.9 ± 3.2
Na^+^ (mMol L^−1^)	GIM	137 ± 1	137 ± 1	137 ± 2	138 ± 2	137 ± 1	138 ± 0
	GIN	139 ± 2	138 ± 1	137 ± 2	138 ± 1	137 ± 1	137 ± 2
K^+^ (mMol L^−1^)	GIM	3.8 ± 0.2	3.8 ± 0.3	3.7 ± 0.1	3.7 ± 0.2	3.8 ± 0.2	3.7 ± 0.3
	GIN	3.6 ± 0.2	3.6 ± 0.2	3.6 ± 0.3	3.6 ± 0.4	3.6 ± 0.5	3.5 ± 0.3
Ca^2+^ (mMol L^−1^)	GIM	1.29 ± 0.04	1.25 ± 0.06	1.25 ± 0.04	1.25 ± 0.06	1.25 ± 0.03	1.25 ± 0.05
	GIN	1.30 ± 0.07	1.29 ± 0.05	1.24 ± 0.06	1.25 ± 0.07^†^	1.29 ± 0.05	1.27 ± 0.09
Cl^−^ (mMol L^−1^)	GIM	103 ± 1	104 ± 1	104 ± 2	104 ± 2	104 ± 2	104 ± 1
	GIN	104 ± 1	103 ± 1	104 ± 1	104 ± 1	103 ± 1	103 ± 1
Glic (mMol L^−1^)	GIM	5.4 ± 0.7	5.7 ± 1.4	5.8 ± 1.6	5.6 ± 1.7	5.5 ± 1.7	5.2 ± 1.1
	GIN	5.3 ± 0.7	5.2 ± 0.8	5.1 ± 1.1	5.0 ± 0.8	5.2 ± 1.0	5.3 ± 1.3
Lac (mMol L^−1^)	GIM	2.3 ± 1.2	2.4 ± 1.0	2.6 ± 1.2	2.9 ± 1.6	3.2 ± 1.8	2.7 ± 1.4
	GIN	2.2 ± 0.8	2.5 ± 1.5	2.7 ± 1.9	3.4 ± 2.0	3.9 ± 2.6	4.6 ± 2.9

^†^Different from T_10_, in the same group, by Dunnett's test (*p* < 0.05). ^*^Minutes after AMK administration. T_rec_: Trecumbency.

**Table 5:** Percentage representation of gasometric parameters of adult pigs, which received a combination of azaperone (3 mg kg^−1^), midazolam (0.3 mg kg^−1^), and ketamine (7 mg kg^−1^), via intramuscular (GIM, *n* = 8) and intranasal (GIN, *n* = 8) routes.

**Variable**	**Time**	**GIN (% of animals)**			**GIM (% de animais)**			**Reference**
		**Below**	**Normal**	**Above**	**Below**	**Normal**	**Above**	**(unit)**
${\text{PaCO}}_{2}^{\text{t}}$	${\text{T}}_{10}^{*}$	0	40	60	0	33	67	^a^35–44 (mmHg)
	T_20_	0	50	50	0	43	57	
	T_30_	0	67	33	0	29	71	
	T_45_	0	38	63	0	29	71	
	T_60_	14	43	43	0	43	57	
	T_90_	20	60	20	0	50	50	
	**Mean**	**5**	**50**	**45**	**0**	**38**	**62**	
${\text{PaO}}_{2}^{\text{t}}$	${\text{T}}_{10}^{*}$	33	67	0	50	50	0	^b^82–108 (mmHg)
	T_20_	25	75	0	67	33	0	
	T_30_	0	75	25	33	67	0	
	T_45_	0	100	0	67	33	0	
	T_60_	0	100	0	33	67	0	
	T_90_	0	100	0	33	33	33	
	**Mean**	**11**	**83**	**6**	**47**	**47**	**6**	
BE	${\text{T}}_{10}^{*}$	40	60	0	33	67	0	^c^4.6–12.6 (mMol L^−1^)
	T_20_	38	63	0	43	57	0	
	T_30_	33	67	0	43	57	0	
	T_45_	38	63	0	43	57	0	
	T_60_	43	57	0	43	57	0	
	T_90_	40	60	0	33	67	0	
	**Mean**	38	62	0	41	59	0	
K^+^	${\text{T}}_{10}^{*}$	100	0	0	67	33	0	^d^3.9–7.8 (mMol L^−1^)
	T_20_	88	13	0	71	29	0	
	T_30_	89	11	0	100	0	0	
	T_45_	75	25	0	71	29	0	
	T_60_	71	29	0	71	29	0	
	T_90_	100	0	0	67	33	0	
	**Mean**	**86**	**14**	**0**	**76**	**24**	**0**	
Ca^2+^	${\text{T}}_{10}^{*}$	100	0	0	100	0	0	^e^2.25–3.1 (mMol L^−1^)
	T_20_	100	0	0	100	0	0	
	T_30_	100	0	0	100	0	0	
	T_45_	100	0	0	100	0	0	
	T_60_	100	0	0	100	0	0	
	T_90_	100	0	0	100	0	0	
	**Mean**	**100**	**0**	**0**	**100**	**0**	**0**	
Glic	${\text{T}}_{10}^{*}$	**0**	100	**0**	**0**	**100**	**0**	^f^2.6–8.6 (mMol L^−1^)
	T_20_	0	100	0	0	100	0	
	T_30_	0	100	0	0	86	14	
	T_45_	0	100	0	0	86	14	
	T_60_	0	100	0	0	86	14	
	T_90_	0	100	0	0	100	0	
	**Mean**	0	**100**	0	0	92	8	
Lac	${\text{T}}_{10}^{*}$	0	20	80	0	33	67	^g^0.5–1.5 (mMol L^−1^)
	T_20_	0	25	75	0	0	100	
	T_30_	0	22	78	0	29	71	
	T_45_	0	13	88	0	29	71	
	T_60_	0	43	57	0	29	71	
	T_90_	0	0	100	0	33	67	
	**Mean**	**0**	**21**	**79**	**0**	**24**	**76**	
pH_st_	${\text{T}}_{10}^{*}$-T_90_	0	100	0	0	100	0	^h^7.4–7.54
${\text{cHCO}}_{3\text{st}}^{-}$	${\text{T}}_{10}^{*}$-T_90_	0	100	0	0	100	0	^i^22–36.6 (mMol L^−1^)
Na^+^	${\text{T}}_{10}^{*}$-T_90_	0	100	0	0	100	0	^j^129–156 (mMol L^−1^)
Cl^−^	${\text{T}}_{10}^{*}$-T_90_	0	100	0	0	100	0	^k^93–126 (mMol L^−1^)

^a^${\text{PaCO}}_{2}^{\text{t}}$: 35–44 mmHg (36); 36–44 mmHg (35). ^b^${\text{PaO}}_{2}^{\text{t}}$: 82–102 mmHg (35). ^c^BE: 4.6–12.6 mMol L^−1^ (35); 7.5–mMol L^−1^ (37). ^d^K^+^: 3.9–4.1 mMol L^−1^ (36); 4.8–7.8 mMol L^−1^ (38). ^e^Ca^2+^: 2.25–2.8 mMol L^−1^ (36); 2.5–3.1 mMol L^−1^ (38). ^f^Glic: 2.6–6.5 mMol L^−1^ (36); 4.3–8.6 mMol L^−1^ (38). ^g^Lac: 0.5–8.5 mMol L^−1^ (36); 2.8–3.1 mMol L^−1^ (37); ^h^pH_st_: 7.4–7.53 (36); 7.45–7.54 (35). ^i^${\text{cHCO}}_{3\text{st}}^{-}$: 22–33 mMol L^−1^ (36); 27.8–36.6 mMol L^−1^ (35). ^j^Na^+^: 129–143 mMol/L (36); 143–156 mMol L^−1^ (38). ^k^ Cl^−^: 93–126 mMol L^−1^ (36); 99.5–112.3 mMol L^−1^ (38). ^*^Minutes after AMK administration. T_rec_: Trecumbency.

BE values did not vary between groups or timepoints but remained below physiological limits in 38% and 41% of GIN and GIM animals, respectively. Lactate values remained consistent between groups and times (Table 4) but exceeded physiological limits in both groups, with an average occurrence of hyperlactatemia of 79% in GIN and 76% in GIM.

Ca^2+^ values did not differ between groups (Table 4) but decreased at T45 in GIN compared to T10 (*p* = 0.0265). As displayed in Table 5, both groups exhibited a high percentage of hypocalcemia (Ca^2+^ < 2.25 mMol/L) and hypokalemia (K^+^ < 3.9 mMol/L).

## 4 Discussion

### 4.1 Characteristics of the chemical restraint

Several studies have explored the intranasal administration of azaperone (13, 14, 21, 27), midazolam (22), and ketamine (13, 14, 27) for CR in pigs, either alone or in combination. These studies reveal variations in latency, duration, and restraint characteristics. Discrepancies can be attributed to variables including the combination of medications, doses, the presence of nociceptive stimuli, and the age/weight of the animals.

A study closely resembling AMK was conducted by (13), using ketamine (15 mg kg^−1^), azaperone (1 mg kg^−1^), and climazolam (1.5 mg kg^−1^) to restrain piglets aged 4 to 7 days. Latency periods ranged from 4 to 5 min, longer than those observed in our study. This difference may be attributed to the animals' weight. Considering a weight-based dosing regimen, it's essential to acknowledge that finishing pigs have a higher fat percentage, resulting in a relatively higher dose for a 110 kg animal than a 2.3 kg animal. In both studies, intranasal administration led to shorter CR durations, emphasizing the effectiveness of this route for procedures that require CR of short and medium duration.

Intranasal administration is characterized by the high permeability of the nasal mucosa, proximity to the central nervous system (31, 32), and facilitated drug absorption through trigeminal and olfactory pathways, avoiding the blood-brain barrier (26). This explains the substantially shorter latency period observed in GIN compared to GIM. The rapid onset of relaxation is a notable advantage, especially considering the swine's susceptibility to stress.

The AMK combination offers advantages over other protocols. Azaperone alone, administered intranasally and intramuscularly to piglets, induced decubitus after 30 and 90 min, respectively (21). Alfaxalone, given intranasally, did not achieve sufficient sedation in adult pigs for handling or clinical procedures (10). Another study showed that S (+) ketamine, in combination with azaperone, did not provide better anesthetic quality than racemic ketamine when administered intranasally (27). These findings underscore the synergistic effect of the combination used in our study, ensuring effective CR regardless of the administration route. The immediate muscle relaxation in GIN animals may be linked to the shorter latency period of drug action in this group.

It's crucial to highlight that the AMK combination provided effective CR and muscle relaxation in the absence of nociceptive stimuli. In Axiak et al.'s study, the azaperone, climazolam, and ketamine combination did not eliminate pain scores during piglet castration, particularly when administered intranasally (13). The substantial final solution volume is one of the limitations of the intranasal route, leading to drug swallowing and compromised absorption due to the first-pass effect (13). Our test involved adult pigs, and the AMK solution had considerable final volumes (21 ± 3.5 mL). Although we did not observe large variations in sedation quality among GIN individuals, formulating more concentrated preparations for intranasal administration, coupled with aerosol-generating spray devices, could be highly advantageous.

### 4.2 Physiological and gasometric parameters

Interpreting physiological variables poses a consistent challenge, particularly given the unique aspects of the swine species. When reviewing literature, considering study-specific factors, including age, genetic lineage, and environmental conditions, is crucial. Caution is warranted in making comparisons and establishing normal or abnormal values. To address this, we adopted a descriptive approach (Tables 3, 5) to complement inferential analyses. Reference values for each parameter were standardized using selected studies, prioritizing similar methodologies (33–38).

The elevated PR at Trec was probably a result of physical restraint. Table 3 reveals a higher incidence of tachycardia than bradycardia in both groups, which could reflect systemic effects induced by the sedative drugs. In individuals with normal serum catecholamine levels, ketamine's sympathomimetic effect increases heart rate, cardiac output, contractile strength, and arterial pressures (18). Additionally, azaperone, through α1-adrenergic blockade, lowers systemic vascular resistance, compensated by an increased heart rate (39).

Only GIN animals exhibited an increased *f*_R_. Typically, the drugs used have minimal impact on the respiratory system (19). These effects, if present, tend to be more associated with respiratory depression (39, 40), except for azaperone, which elevated *f*_R_ in rats, horses, and pigs (41). The intranasal route may account for the observed differences in *f*_R_ between groups and the decreased SpO_2_ in GIN at Trec. Slow administration aimed to minimize swallowing and drug entry into the lower respiratory tract, but no method ensured liquid confinement to nasal and tracheal mucosa. Consequently, it is believed that AMK contact with lung epithelium may transiently reduce SpO_2_, leading to tachypnea in GIN animals at T45, T60, and T90.

It is crucial to emphasize that the shed temperature was regulated, and the sedation durations for the animals were standardized, thereby minimizing the impact of external factors on Trectal. Consequently, we can infer that the thermoregulation system of GIN animals experienced less disruption compared to GIM animals, supporting the findings of Axiak et al. (13).

Regarding acid-base balance, the observed base deficit in both groups may be linked to elevated lactate levels, possibly induced by physical restraint. A study by Hamilton et al. noted lactate levels of 4.9 mMol/L in pigs under light handling and 19.1 mMol/L in those subjected to heavy handling (37). Nevertheless, it is crucial to emphasize that the rise in lactate did not induce alterations in pH and serum bicarbonate levels. This implies that primary buffering systems effectively contributed to maintaining homeostasis in both groups.

The hypocapnia noted in certain GIN animals at T60 and T90 aligns with the concurrent increase in respiratory rate during these intervals in this group. Hyperventilation may perhaps justify the absence of hypoxia in GIN between T45 and T90, unlike GIM, in which 47% and 33% of animals had ${\text{PaO}}_{2}^{\text{t}}$ values lower than 82 mmHg, at T45 and T90, respectively (Table 5).

Depletion of potassium and calcium can be ascribed to fasting. A prior study involving crossbred pigs weighing around 30 kg showed no significant potassium level variations after a period of food deprivation (42). However, this study noted a decrease in calcium levels only after prolonged fasting (51, 95, and 167 h), contrasting with our observations of hypokalemia and hypocalcemia in animals with significantly higher weights subjected to an 8-h fast. Our findings align with Becerril-Herrera et al. (43), who assessed biochemical and gasometric parameters in Pietran, Large White, and Yorkshire pigs designated for slaughter. They observed a reduction in the levels of these two electrolytes after 8 and 16 h of food deprivation (43), suggesting that weight and bloodline may influence the metabolic response during fasting.

## 5 Conclusions

Intranasal administration of the AMK combination produced immediate chemical restraint, with quality similar to that obtained after intramuscular administration, with minimal clinical changes. The latency and duration of the effect make the intranasal route a suitable option for carrying out usual ambulatorial or surgical procedures, in association with local anesthesia. Furthermore, given the association of many adverse effects of anesthesia with prolonged recovery periods, the use of this route suggests a reduction in the incidence of these effects compared to intramuscular administration.

## Data Availability

The raw data supporting the conclusions of this article will be made available by the authors, without undue reservation.

## Supplementary material

Supplementary Video 1 Intranasal administration of azaperone, midazolam, and ketamine (AMK) combination, over 20 seconds, in an adult male pig.
